# Chinese pediatric Tuina can prevent premature infant feeding intolerance and is conducive to weight gain: a prospective randomized controlled study

**DOI:** 10.4314/ahs.v23i2.80

**Published:** 2023-06

**Authors:** Shao-Shu Li, Xiu-Yao Lin, Xin Li, Ya-Di Zhang, Li-Qiong Wang, Su-xian Lai

**Affiliations:** Department of Pediatrics, Quanzhou First Hospital Affiliated to Fujian Medical University

**Keywords:** Chinese pediatric *Tuina*, wremature infant, feeding intolerance, weight gain, nutritional status

## Abstract

**Objective:**

Preterm birth is one of the most important health problems in the world. Feeding intolerance is one of the most common and serious complications of premature infant. The purpose of this study was to explore the effect of Chinese pediatric Tuina on the prevention of feeding intolerance in favour of weight gain in premature infants.

**Methods:**

A prospective randomized controlled study was conducted in the Department of Neonatology in our hospital. Premature infants were recruited and randomly assigned to an intervention group or a control group. Premature infants in the intervention group received a Chinese pediatric Tuina intervention by professional chiropractors, while premature infants in the control group received standard care. The incidence of feeding intolerance and weight gain situation were compared between the two groups.

**Result:**

After 1 week of intervention, the body weight (2.5±0.5 vs 2.0±0.4, p=0.038), head circumference (32.8±1.7 vs 29.9±1.4, p=0.041), albumin (34.6±5.8 vs 28.4±6.1, p-0.026) and prealbumin (155.8±35.2 vs 113.6±36.8, p=0.021) of preterm infants in the intervention group were significantly better than those in the control group. The incidence of feeding intolerance (7 vs 15, p=0.032) in the intervention group was significantly lower than that in the control group. Although there were no statistically significant differences (P>0.05), the incidences of gastrointestinal bleeding, necrotizing enterocolitis, and liver insufficiency were lower in the intervention group than in the control group.

**Conclusion:**

Chinese pediatric Tuina can effectively prevent the occurrence of feeding intolerance in premature infants and be conducive to the weight gain and improving nutritional status of premature infants.

## Introduction

Preterm birth is one of the most important health problems in the world.^1^,^2^ At present, premature birth is the second most common reason for death among children under five years of age and the most common reason for death during the first month of life.^3^ The incidence of preterm birth is increasing globally, with 5-13% of babies born preterm and 15 million premature babies born every year. Preterm birth has become a serious public health problem worldwide.^4^

Due to their low gestational age and low weight at birth, the immature development of various systems and organs, poor sucking and swallowing function, and insufficient secretion of digestive enzymes, preterm infants are prone to various complications, and feeding intolerance is one of the most common and serious complications.^5^-^7^ Feeding intolerance mainly manifests as vomiting, abdominal distension, difficulty in increasing milk volume, gastric retention, abnormal defecation and so on. The appearance of feeding intolerance in premature infants leads to longer periods of intravenous nutrition, cholestasis, hospital infection, longer hospitalization, and more societal loss. Therefore, the early establishment of adequate gastrointestinal nutrition for premature infants is one of the important conditions for the successful treatment of premature infants.^8^ Chinese pediatric Tuina is a traditional Chinese medicine treatment in China that is non-invasive, convenient and safe and is a special type of massage. Many studies have shown the obvious benefits of massage for premature babies.^9^-^12^ Kadir et al found that abdominal massage can effectively prevent feeding intolerance in premature infants.^13^ Study of Momenfar et al. also showed that massage can promote gastrointestinal motility and make food easier to digest, which can reduce the incidence of feeding intolerance.^14^ A meta-analysis of Wang L showed that massage can reduce gastrointestinal complications and promote weight gain.^15^ To investigate the effect of Chinese pediatric Tuina in preventing feeding intolerance and promoting weight gain in premature infants, a prospective randomized controlled study was conducted in our hospital.

## Method

The present study was approved by the ethics committee of our hospital and adhered to the tenets of the Declaration of Helsinki. Additionally, all the parents of patients signed the consent form before participating in the study.

### Research design

This study was a prospective randomized controlled study conducted in the Department of Neonatology in a hospital on the southeast coast of China from June 2019 to October 2020. Based on the difference in the incidence of feeding intolerance (13% vs 35%) between the two groups from the pre-experiment, and assuming that the alpha value was set at 0.05 with a power of 0.80, the required number of participants was calculated to be 45 in each group. Assuming a 10% rate of missing data, the total sample size was set at 100 (50 per group). A total of 100 Premature infants were enrolled in this study.

The inclusion criteria were as follows: 1) preterm infants who received enteral feeding, 2) a gestational age of 28-36 weeks, 3) vital sign stationarity, 4) no intestinal obstruction or abdominal surgery, 5) no skin disease, and 6) parents' informed consent. The exclusion criteria were as follows: 1) unstable medical condition; 2) complications of severe congenital malformations, such as severe congenital heart disease and gastrointestinal abnormalities, etc.; and 3) weight<1000 g.\

### Recruitment

Participants were recruited according to the inclusion criteria and given written information regarding the study. Informed consent was obtained from all parents of patients. Withdrawal from the study was possible at any time without any negative consequences.

### Randomization

Simple randomization was done according to a software-generated random number sequence posted on a dedicated and secure website available on a 24/7 basis. The website generated the randomization, but the sequence was concealed from investigators. Participants who were eligible were randomized to either the intervention group received Chinese pediatric Tuina intervention by professional chiropractors or the control group received standard care. Patients randomized to one arm could not cross over to the others during the study.

### Standard care

In all preterm infants, orogastric tubes were inserted. A 6 Fr orogastric tube is generally inserted in these infants. Bolus feedings were given by gravity drainage after priming the tubing every three hours in the two groups. Continuous feeds were given using an automatic syringe pump. Gastric residuals were checked every three hours in infants in the two groups, regardless of assignment. In the unit, preterm infants were fed eight meals a day. The feeding of the preterm infants was started with a 2- or 3-ml feeding bolus for the first feeding. As food toleration developed, this amount was increased to 20 ml/kg/ day and continued until it reached 140-160 ml/kg/day. During infant gavage feeding, the flow rate of milk was caused by gravity using the suspension technique without applying pressure to the injector. Body weight was measured and recorded daily before the 9:00 a.m. feeding during the study.^7^

### Chinese pediatric Tuina

The intervention group was treated with Chinese pediatric Tuina on the basis of standard care. Two professionals pediatric Tuina doctors who had 5 years of experience in Chinese pediatric *Tuina* participated in the trial. The method was a follows: 1) Zusanli was kneaded for 3 minutes; 2) the abdomen Yin and Yang was pushed for 5 minutes; 3) the abdomen was massaged for 3 minutes; 4) the lower Qigujie was pushed for 3 minutes; 5) Yuwei was kneaded for 2 minutes; 6) Sanguan was pushed one hundred to three hundred times and Liufu was pushed one hundred to three hundred times for a total of 3 minutes; 7) Hukou was pushed one hundred to three hundred times, Bu Pishi was pushed one hundred to three hundred times, and Yun Bagua was pushed one hundred to three hundred times for a total of 5 minutes; 8) Zhongwan was massaged for 3 minutes; and 9) chiropractic care was provided for 3 minutes. The total time was controlled to be within 30 minutes once a day. One course of treatment lasted for 7 days.

### Data collection

Before the intervention, the following data were collected: gestational age, age, sex, body weight, head circumference, albumin and prealbumin. After one week of intervention, the following data were collected: body weight, head circumference, albumin, prealbumin and complications including feeding intolerance, gastrointestinal bleeding, necrotizing enterocolitis, and liver insufficiency. The diagnostic criteria of feeding intolerance, based on those from American Academy of Pediatrics guidelines, were any of the following: 1) severe abdominal distension; 2) gastric retention ≥ 25%-50% of the total amount of 2-3 feedings; 3) obvious bloody stools; and 4) bile reflux or vomiting.^16^

### Statistical Analysis

We used SPSS 25.0 software for statistical analysis. Continuous data are presented as the mean ± standard deviation and range. Clinical parameters between the two groups were compared with an independent-sample t-test. The χ^2^ or Fisher's test was used for categorical variables. A p value of <0.05 was defined as statistically significant.

## Result

Before intervention, there were no statistically significant differences in gestational age, age, sex, body weight, head circumference, albumin or prealbumin between the two groups, which indicated that the two groups were homogenous and comparable (Table 1).

**Table 1:** Comparison of general data between the two groups

	Intervention group	Control group	P value
Gestational age (weeks)	34.1±3.2	34.4±2.9	0.673
Age (days)	10.8±5.7	12.3±7.2	0.354
Boys/girls	27/23	24/26	0.548
Body weight (kg)	2.1±0.3	1.9±0.3	0.562
Head circumference (cm)	30.1±1.6	29.8±1.3	0.783
Albumin (g/L)	29.8±6.2	27.6±5.5	0.615
Prealbumin (mg/L)	108.7±23.5	102.5±31.7	0.434

After 1 week of intervention, the body weight, head circumference, albumin and prealbumin of preterm infants in the intervention group were significantly better than those in the control group (P<0.05) (Table 2).

**Table 2:** Comparison of the nutritional status between the two groups after one-week intervention

	Intervention group	Control group	P value
Body weight (kg)	2.5±0.5	2.0±0.4	0.038
Head	32.8±1.7	29.9±1.4	0.041
circumference (cm)			
Albumin (g/L)	34.6±5.8	28.4±6.1	0.026
Prealbumin (mg/L)	155.8±35.2	113.6±36.8	0.021

After 1 week of intervention, the incidence of feeding intolerance in the intervention group was significantly lower than that in the control group (14% vs 30%, P= 0.032). Although there were no statistically significant differences (P>0.05), the incidences of gastrointestinal bleeding, necrotizing enterocolitis, and liver insufficiency were lower in the intervention group than in the control group (Table 3).

**Table 3:** Comparison of the complications between the two groups after one-week intervention

	Intervention group	Control group	P value
Feeding intolerance	7	15	0.032
Gastrointestinal bleeding	2	4	0.400
Necrotizing enterocolitis	2	5	0.240
Liver insufficiency	4	7	0.338

## Discussion

Many premature infants are transferred to the neonatal intensive care unit after birth and are fed via a tube (nasal or oral).^17^ Premature infants are prone to feeding intolerance due to immature development, poor function of various organs, the dysplasia of villi on the surface of the gastrointestinal tract, insufficient secretion of various digestive enzymes, and different intestinal microflora, and the premature infants with younger gestational age have more obvious feeding intolerance.^18^,^19^ Feeding intolerance is very common in preterm infants, feeding problems are one of the main factors leading to longer stays of preterm infants in the NICU, and feeding intolerance is also an important factor affecting the weight gain and growth of preterm infants.^6^,^7^ Rapidly reaching adequate feeding is beneficial to the development and weight gain of premature infants, reducing the time of intravenous nutrition, the occurrence of complications such as cholestasis, nosocomial infection, and metabolic disorders, and the length of hospital stay; saving societal and economic costs; and reducing the waste of medical resources.^8^

Studies have shown that direct stimulation of the vagus nerve can regulate gastric peristalsis, enhance food digestion, and increase nutrient utilization.^20^ Insulin-like growth factor-1 plays a key role in regulating the growth of premature infants, and the stimulation of stress receptors increases vagal nerve activity, which promotes insulin release and indirectly leads to insulin-like growth factor-1 release.^21^ Numerous studies have shown that massage was of great benefit to infants. A systematic review study of Seiiedi-Biarag L showed that most studies reported that the administration of various forms of therapeutic massage exerted a beneficial effect on the growth of preterm infants, including increased vagal activity. 2 Study of Field T has shown that massage can stimulate vagus nerve activity, leading to gastrointestinal reactions, and produce mechanical and reflexive effects in the intestinal tract, thereby reducing abdominal distention and increasing intestinal movement.^22^ Diego MA found that massage was related to increased vagal activity which was beneficial to reduce the incidence of feeding intolerance and promote weight gain.^23^ Our study also had the same result, we found that after Chinese pediatric Tuina, the incidence of feeding intolerance of premature infants decreased significantly, and the weight gain was better.

Chinese pediatric *Tuina* is a special type of massage. Tuina is widely used in traditional Chinese medicine physiotherapy and can be used to treat some common conditions, such as pediatric diarrhea, fever and feeding intolerance.^24^ According to meridian theory, these acupoints are associated with specific organs, and by stimulating these specific acupoints, they can help normalize impaired function.^25^ According to traditional Chinese medicine theory, the delicate viscera of premature infants, the slow increase in feeding amount, and even apparent feeding intolerance belong to the syndrome of spleen deficiency, and the pathogenesis lies in the lack of power and food stagnation. The principle of treatment is regulating the spleen and stomach, eliminating the accumulation leading to stagnation. The function of Zang Fu organs can be adjusted by stimulating acupoints with various techniques.^26^ Chinese pediatric Tuina can reflexively increase the activity of the vagus nerve, which can promote the release of gastrointestinal hormones, such as insulin and gastrin; gastrointestinal peristalsis, which promotes the maturity of gastrointestinal digestive function; and the gastrointestinal digestion and absorption of premature infants. Chinese pediatric Tuina is a non-invasive, nondrug clinical treatment and will not increase the burden of the gastrointestinal tract, which has obvious advantages for premature infants in the establishment of sufficient feeding. This study showed that after Chinese pediatric *Tuina* treatment, the weight gain and nutritional status of premature infants in the intervention group were significantly higher than those in the control group, and the incidence of feeding intolerance in the intervention group was significantly decreased compared with that in the control group. In the process of Chinese pediatric *Tuina* treatment, attention should be given to the protection of premature infants. Chinese pediatric Tuina treatment should be performed 1 hour after feeding, and the movements should be gentle. Proper room temperature should be maintained, and strict hand disinfection should be performed. Hand disinfection should be repeated before touching different premature infants to avoid nosocomial infection.

This study has some limitations. The participants were recruited from one hospital, thus reducing the generalizability of these findings.

## Conclusion

Chinese pediatric *Tuina* is a non-invasive clinical treatment method that can effectively prevent the occurrence of feeding intolerance in premature infants and be conducive to the weight gain and improving nutritional status of premature infants. Although this study provided scientific evidence for the advantages of Chinese pediatric Tuina, more research is necessary.
